# Internet-Based Multimodal Pain Program With Telephone Support for Adults With Chronic Temporomandibular Disorder Pain: Randomized Controlled Pilot Trial

**DOI:** 10.2196/22326

**Published:** 2020-10-13

**Authors:** Julia Lam, Peter Svensson, Per Alstergren

**Affiliations:** 1 Department of Orofacial Pain and Jaw Function Faculty of Odontology Malmö Sweden; 2 Folktandvården Skåne AB Hässleholm and Lund Sweden; 3 Scandinavian Center for Orofacial Neurosciences (SCON) Malmö Sweden; 4 Scandinavian Center for Orofacial Neurosciences (SCON) Aarhus Denmark; 5 Department of Orofacial Pain and Jaw Function Faculty of Health Aarhus University Aarhus Denmark; 6 Specialized Pain Rehabilitation Skåne University Hospital Lund Sweden

**Keywords:** chronic pain, cognitive behavior therapy, combined modality therapy, facial pain, feasibility studies, health services research, internet-based intervention, occlusal splints, pilot projects, temporomandibular disorders

## Abstract

**Background:**

Chronic pain from temporomandibular disorders remains an undertreated condition with debate regarding the most effective treatment modalities.

**Objective:**

The aim of the study was to investigate the treatment effect of an internet-based multimodal pain program on chronic temporomandibular disorder pain and evaluate the feasibility of a larger randomized controlled trial.

**Methods:**

An unblinded randomized controlled pilot trial was conducted with 43 participants (34 females, 9 males; median age 27, IQR 23-37 years) with chronic temporomandibular pain. Participants were recruited within the Public Dental Health Service and randomized to intervention (n=20) or active control (n=23). The intervention comprised a dentist-assisted internet-based multimodal pain program with 7 modules based on cognitive behavior therapy and self-management principles. The control group received conventional occlusal splint therapy. Primary outcomes included characteristic pain intensity, pain-related disability, and jaw functional limitation. Secondary outcomes were depression, anxiety, catastrophizing, and stress. Outcomes were self-assessed through questionnaires sent by mail at 3 and 6 months after treatment start. Feasibility evaluation included testing the study protocol and estimation of recruitment and attrition rates in the current research setting.

**Results:**

Only 49% of participants (21/43) provided data at the 6-month follow-up (internet-based multimodal pain program: n=7; control: n=14). Of the 20 participants randomized to the internet-based multimodal pain program, 14 started treatment and 8 completed all 7 modules of the program. Between-group analysis showed no significant difference for any outcome measure at 3- or 6-month follow-up—characteristic pain intensity (3 months: *P*=.58; 6 months: *P*=.41), pain-related disability (3 months: *P*=.51; 6 months: *P*=.12), jaw functional limitation (3 months: *P*=.45; 6 months: *P*=.90), degree of depression (3 months: *P*=.64; 6 months: *P*=.65), anxiety (3 months: *P*=.93; 6 months: *P*=.31), stress (3 months: *P*=.66; 6 months: *P*=.74), or catastrophizing (3 months: *P*=.86; 6 months: *P*=.85). Within-group analysis in the internet-based multimodal pain program group showed a significant reduction in jaw functional limitation score at the 6-month follow-up compared to baseline (Friedman: χ2=10.2, *P*=.04; Wilcoxon: z=–2.3, *P*=.02). In the occlusal splint group, jaw function limitation was also reduced at the 6-month follow-up (Friedman: χ2=20.0, *P*=.045; Wilcoxon: z=–2.3, *P*=.02), and there was a reduction in characteristic pain intensity at the 3- and 6-month follow-up (Friedman: χ2=25.1, *P*=.01; Wilcoxon 3 months: z=–3.0, *P*=.003; Wilcoxon 6 months: z=-3.3, *P*=.001).

**Conclusions:**

This study was not able to demonstrate a difference in treatment outcome between an internet-based multimodal pain program and occlusal splint therapy in patients with chronic temporomandibular pain. However, the findings suggested that the internet-based multimodal pain program improves jaw function. The results also confirmed the treatment effect of occlusal splint therapy for chronic temporomandibular pain. Furthermore, because of the high attrition rate, this pilot study showed that a randomized controlled trial with this design is not feasible.

**Trial Registration:**

ClinicalTrials.gov NCT04363762; https://clinicaltrials.gov/show/NCT04363762

## Introduction

Temporomandibular disorders (TMD) are underdiagnosed and undertreated conditions in the general population [1]. TMD involves conditions affecting the temporomandibular joint and the masticatory muscles and has a prevalence of 10%-15% in the adult population [2]. Typical TMD complaints consist of pain, fatigue, and stiffness in the jaw muscles; limitations in jaw movements; and clicking or grating noises from the jaw joints [3]. Chronic TMD pain negatively affects quality of life, and in Sweden, even though the estimated treatment need is 5%-15%, only 0.5%-1.5% receive treatment in general dentistry [4,5]. The Swedish national guidelines [1] recommend a multimodal treatment approach, and common treatment options include occlusal appliances, physiotherapy, pharmacologic treatment, and behavior treatment. There are still debates regarding the most efficient and cost-effective treatment option that can easily be distributed and applied.

Internet-based intervention is an appealing modality for multimodal TMD treatment as it may not only assist dentists in providing treatment at reduced costs but may also potentially reduce traveling time, treatment costs, and waiting lists for patients. Generally, in the field of pain, there are several apps for various chronic pain conditions (eg, headache, fibromyalgia, and back pain) [6-9]. Internet-based treatment reduces pain intensity and pain interference [9,10] and internet-based cognitive behavior therapy seems to be at least as effective as a face-to-face intervention for chronic pain [11]. However, findings regarding depression and anxiety are inconsistent [12], and there are no internet-based cognitive behavior therapy studies on chronic TMD pain.

This study set out to collect data on the effectiveness of a guided internet-based multimodal pain program for adults with chronic TMD pain compared to that of conventional occlusal splint therapy. As the study progressed, it also became a measure to evaluate the feasibility of a larger randomized controlled trial.

## Methods

### Study Design

An unblinded, parallel-arm, pilot randomized controlled trial with equal allocation was conducted. The participants were randomized to the internet-based multimodal pain program or to an active control. The study was approved by the Ethics Review Board in Lund, Sweden (No. 2016/6). The examination and treatments were free of charge for the participants, and no other financial compensation was given. See Multimedia Appendix 1 for the informed consent documentation in Swedish. This study was retrospectively registered at ClinicalTrials.gov (NCT04363762).

Furthermore, this study was part of a large research project with a primary objective of studying changes in the brain in patients with chronic TMD pain after treatment. Therefore, all participants underwent magnetic resonance imaging (MRI) of the brain. The neuroimaging data will be presented elsewhere; however, the feasibility aspect of this additional examination will be addressed here.

### Participants

Participant inclusion criteria were (1) age between 18 and 75 years; (2) at least one TMD pain diagnosis such myalgia, myofascial pain with referral, headache attributed to TMD, or arthralgia according to the Diagnostic Criteria for Temporomandibular Disorders (DC/TMD) [13]; (3) chronic (≥3 months) TMD pain, experienced once a week or more often, with an intensity of ≥3 (on a scale from 0 to 10); (4) access to a computer with an internet connection and a mobile phone; (5) sufficient computer literacy; and (6) Swedish language fluency. Exclusion criteria were (1) chronic inflammatory systemic diseases; (2) all psychiatric disorders except depression and anxiety due to high comorbidity; (3) occlusal splint therapy in the past 12 months; (4) ongoing extensive dental treatment; and (5) conditions precluding MRI examination.

### Procedure

Participants were recruited from a general dental care clinic (Fäladstorget, Lund) within the National Dental Care in Skåne, Sweden. To identify potential participants, all consecutive adult patients visiting for a regular dental check-up were screened with the 2Q/TMD screening tool [14,15]. If a patient was deemed eligible for the inclusion examination, oral and written information about the study were given, and a new appointment was booked for an inclusion examination. The patient was asked to provide written informed consent, if they were deemed eligible after the inclusion examination. Permuted block randomization with a fixed block size of 10 was used. At the moment of assignment, the local research coordinator blindly picked a piece of paper from an envelope with allocated treatment. Before the study start, 6 sets of opaque envelopes with 10 allocation notes each, 5 with “internet-based multimodal pain program” and 5 with “occlusal splint,” were prepared. All 10 notes in one envelope had to be used before the next envelope was opened. If allocated to internet-based multimodal pain program, working material was sent to the participant by mail, and the treatment was started, with assistance provided by phone. If allocated to treatment with occlusal splint, a new appointment was booked for delivering the splint. Follow-up was performed 3 and 6 months after treatment start with questionnaires that were mailed to the participants. Participants also underwent an MRI of the brain at baseline, after randomization but before treatment start, and 6 months after treatment start at Skåne University Hospital, Malmö, Sweden.

Participant recruitment started in April 2016 and ended in December 2018. Screening was paused during annual summer holiday periods (June-August 2016, 2017, 2018) and during February-April 2017 due to technical issues with the MRI scanner.

### Inclusion Examination

All participants underwent a standardized examination according to DC/TMD to control for eligibility and collect baseline data. The DC/TMD includes a clinical examination and questionnaires described below [13]. Demographic data and pretreatment expectancies on allocated treatment were also assessed.

### Clinical Examination

The clinical DC/TMD examination comprises a standardized assessment of pain and headache locations, jaw opening capacity, pain on mandibular movement, pain on palpation, and temporomandibular joint noises [13]. The clinical examinations were carried out by one of 2 trained dentists [16].

The DC/TMD Symptom Questionnaire was used to assess pain symptoms involving the jaw, jaw noise and locking, and headache. Data from this questionnaire were combined with findings from the clinical examination for diagnosis according to DC/TMD [13].

### Questionnaires

#### Pain

The Graded Chronic Pain Scale [17] was used to assess the subdomains of characteristic pain intensity (pain intensity for reported worst, current, and average pain) and pain-related disability (how much facial pain changed the patient's ability to participate in daily activities, social activities, and work). The questionnaire included the assessment of pain intensity and pain-related disability scored on a 0-10 numeric rating scale, modified from [17].

A full-body pain drawing was used to assess pain locations and distribution [18]. Pain distribution was categorized as local pain in the face area; regional pain in the neck area in addition to local pain; or widespread pain at any other site of the body in addition to local or regional pain. 

#### Physical Functioning

The Jaw Functional Limitation Scale-8 was used to assess jaw function in the masticatory system [19]. The Oral Behaviors Checklist was used to assess the self-reported frequency of oral parafunctional behaviors during awake time [20].

#### Emotional Functioning

The Patient Health Questionnaire-9 was used to assess depression, and the Patient Health Questionnaire-15 was used to assess nonspecific physical symptoms [21,22]. Anxiety was assessed with the Generalized Anxiety Disorders-7 [23], stress was assessed with the Perceived Stress Scale-10, and catastrophizing was assessed with the Pain Catastrophizing Scale [24,25].

#### Patient Outcome Expectancy

A Swedish translation of the Stanford Expectations of Treatment Scale was used to assess positive and negative pretreatment expectancies of treatments [26].

### Treatment Modalities

#### Intervention

The internet-based multimodal pain program is based on cognitive behavior therapy and self-management principles that help patients to cope with chronic TMD pain. It was developed for use in general dentistry by orofacial pain specialists at the Department of Orofacial Pain and Jaw Function at Malmö University, Sweden, in collaboration with Psykologpartners AB (a Swedish provider of internet-based psychological treatment).

**Figure 1:**
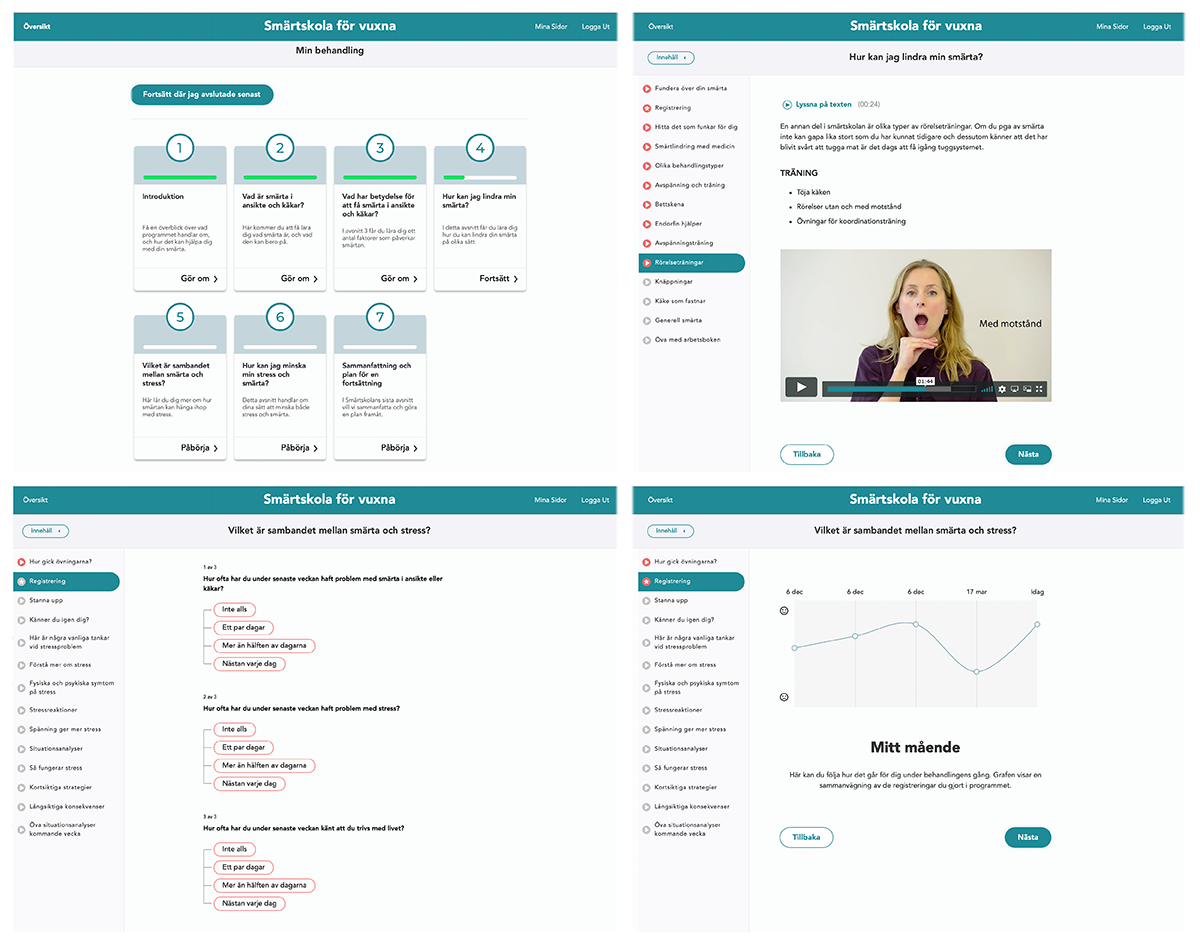
Screenshots from the guided internet-based multimodal pain program (top left: start page; top right: a page with a jaw exercises instructional video; lower left: a page with the recurrent rating of pain frequency, stress level, and life satisfaction to keep track of progression; lower right: an overview of treatment progression).

The internet-based multimodal pain program adapts face-to-face therapy to a software platform program. Access required a 2-factor authentication system. A demo of the program is available (Multimedia Appendix 2). The internet-based multimodal pain program consisted of 7 treatment modules (Table 1, Figure 1) and offline activities included homework assignments in a paperback workbook. The intended treatment duration was 7 weeks: 1 module/week; 40 minutes/module online plus time for homework assignments.

The internet-based multimodal pain program was designed to be used without guidance. However, to support study adherence, telephone, email, and asynchronous chat support was provided by a dentist (JL) who had received 2-day training on internet-based cognitive behavior therapy arranged by Psykologpartners AB. At the start-up phone call, participants were guided through the functions in the program and were informed about time requirements for the treatment. Follow-up phone calls after each finished module involved individualized support and feedback. If participants were unreachable at scheduled follow-up calls, a message through the chat function in the program was sent with a prompt to get in touch. If still not heard from in the subsequent 2 weeks, the dentist tried to contact the participant by phone. If still not reached, access to the internet-based multimodal pain program was removed, and the participant was withdrawn from the study. A letter was sent to the participant with information about the withdrawal and a prompt to seek treatment from a dentist.

**Table 1:** An overview of the internet-based multimodal pain program for chronic temporomandibular disorders pain.

Module	Theme	Content	Cognitive behavior therapy component
1	Introduction	Information: introduction to the treatment program and cognitive behavior therapy, goal setting Assignment: a reflection on previous strategies of handling temporomandibular disorder pain, assessment of core values	Values and goals
2	What is face and jaw pain?	Information: etiology and epidemiology of temporomandibular disorder pain, anatomy of the masticatory system Assignment: pain drawing, identify jaw functions limited by pain	Psychoeducation and assessment
3	What affects my pain in the face and jaw?	Information: modulating and maintaining factors of temporomandibular disorder pain, pain physiology, acute and chronic pain, the link between quality of life and temporomandibular disorder pain Assignment: 7-day pain diary	Psychoeducation and assessment
4	How can I relieve my pain?	Information: treatment alternatives including analgesics, relaxation, jaw exercises, different occlusal splints, acupuncture, and massage Assignment: identify pain modulators from pain diary, practice relaxation, and jaw exercises	Applied relaxation and skills training
5	The relation between stress and pain	Information: stress responses, the link between stress and pain Assignment: a questionnaire to assess the degree of stress, situational analyses, list desired ways to enhance recuperation, continue relaxation and jaw exercises	Functional analysis, problem-solving, relaxation, skills training
6	How can I reduce my stress and pain levels?	Information: stress and pain management; breathing exercises, diet, sleep, training, time management, setting boundaries and acceptance, continued relaxation and jaw exercises Assignment: mapping sleep, training and dietary habits in a weekly schedule and do an activity planning aiming to make desired changes regarding these, continued relaxation and jaw exercises	Committed action, relaxation, skills training
7	Summary and a plan for future action	Information: Setbacks and maintenance planning, a summary of the program Assignment: Develop a maintenance plan	Summary and maintenance plan

#### Control Condition

Participants in the active control group received a hard Michigan-type stabilization splint placed in the upper jaw [27]. The occlusal splint was chosen as the control treatment because it is a conventional and reversible treatment for TMD pain, and it has known moderate efficacy [5]. The splints were made of acrylic, covered all the maxillary teeth, and had a smooth, flat surface. It was fitted to allow all supporting teeth to contact simultaneously on jaw closure, and canine guidance and disocclusion of the posterior teeth were provided in the lateral and protrusive excursion. Participants were instructed to wear it only at night. A clinical check-up of the participants in the occlusal splint group was performed within 2-6 weeks after treatment start. Scheduled time at the dentist was in total 60 minutes for this therapy.

#### Follow-up

Follow-up was conducted with questionnaires comprising a short version of the DC/TMD instruments. The questionnaires were sent to participants by mail at 3 and 6 months after treatment start. If the questionnaire was not returned within a month, a reminder was sent by email or by phone.

### Outcome Measures

#### Preliminary Evaluation of Participant Responses to Intervention

Measures specific to TMD included in the DC/TMD were used as outcome measures, guided by the Initiative on Methods, Measurement, and Pain Assessment in Clinical Trials (IMMPACT) recommendations on core domains [28,29]. Primary outcomes included characteristic pain intensity, pain-related disability, and physical functioning (jaw functional limitation score). Secondary outcomes were depression and anxiety as measures of emotional functioning. The IMMPACT core domains also include ratings of global improvement and satisfaction with treatment, but this was not assessed in this study. In addition to the IMMPACT core domains, catastrophizing and stress were evaluated as secondary outcomes. 

#### Feasibility

Feasibility evaluation of the study included testing the study protocol and estimating recruitment and attrition rates in the current research setting. With preliminary results, post hoc power and sample size calculation were performed. Recruitment and attrition rates along with a new estimated sample size allowed a decision on the feasibility of conducting a future larger randomized controlled trial to be made. Usage metrics of internet-based multimodal pain program were not assessed.

#### Adverse Effects

Adverse events for the internet-based multimodal pain program treatment were collected through the follow-up phone calls. Also, all participants randomized to internet-based multimodal pain program were given written information at the 3-month follow-up about the opportunity to withdraw from the study and receive rescue treatment if symptoms worsened or if they were not satisfied with the treatment outcome. In a similar manner, participants randomized to occlusal splint therapy were free to withdraw from the study at any time, without penalty or loss of benefits, to seek additional treatment.

### Statistical Analysis

Nonparametric statistics were used due to the characteristics of the pain-related variables. For descriptive statistics, median, 25th percentile, and 75th percentile, as well as the number of observations or proportions, are reported. To explore the potential effects of the internet-based multimodal pain program in this early-stage trial, per protocol between-group comparisons of change in characteristic pain intensity level, pain-related disability, jaw functional limitation score, degree of depression, anxiety, catastrophizing, and stress at the 3- and 6-month follow-up were performed with the Wilcoxon rank-sum test. Friedman analysis of variance was used for within-group comparisons. Significant results from the Friedman analysis were followed by posthoc Wilcoxon matched-pair signed-rank testing. In addition, Spearman rank-order was used to test possible association between positive and negative treatment expectancy and changes in outcome measures at the 6-month follow-up within respective treatment groups. Dropout analysis was performed to test the difference between completers and dropouts within allocated treatment groups. Furthermore, a comparison of baseline characteristics of participants between study groups was made. Chi-square test was used for categorical variables and the Wilcoxon rank-sum test was used for ordinal variables.

Estimation of required sample size was based on a previous neuroimaging study to detect statistically significant results in functional MRI [30]. According to this, 20 participants in each group should be recruited; however, the goal was to recruit 30 participants in each group to compensate for dropouts. It was also considered to be sufficient to provide useful information about the feasibility of a future larger randomized controlled trial. A posthoc power analysis with characteristic pain intensity as the outcome measure was performed. The sample size required for a parametric test on the between-group comparison was computed, and 15% was added, as nonparametric testing could be used in a future randomized controlled trial [31].

All data management and statistical analyses were performed unblinded using StataSE (version 15.1; StataCorp LLC). A probability level of *P<*.05 was considered significant.

## Results

### Participants

**Figure 2:**
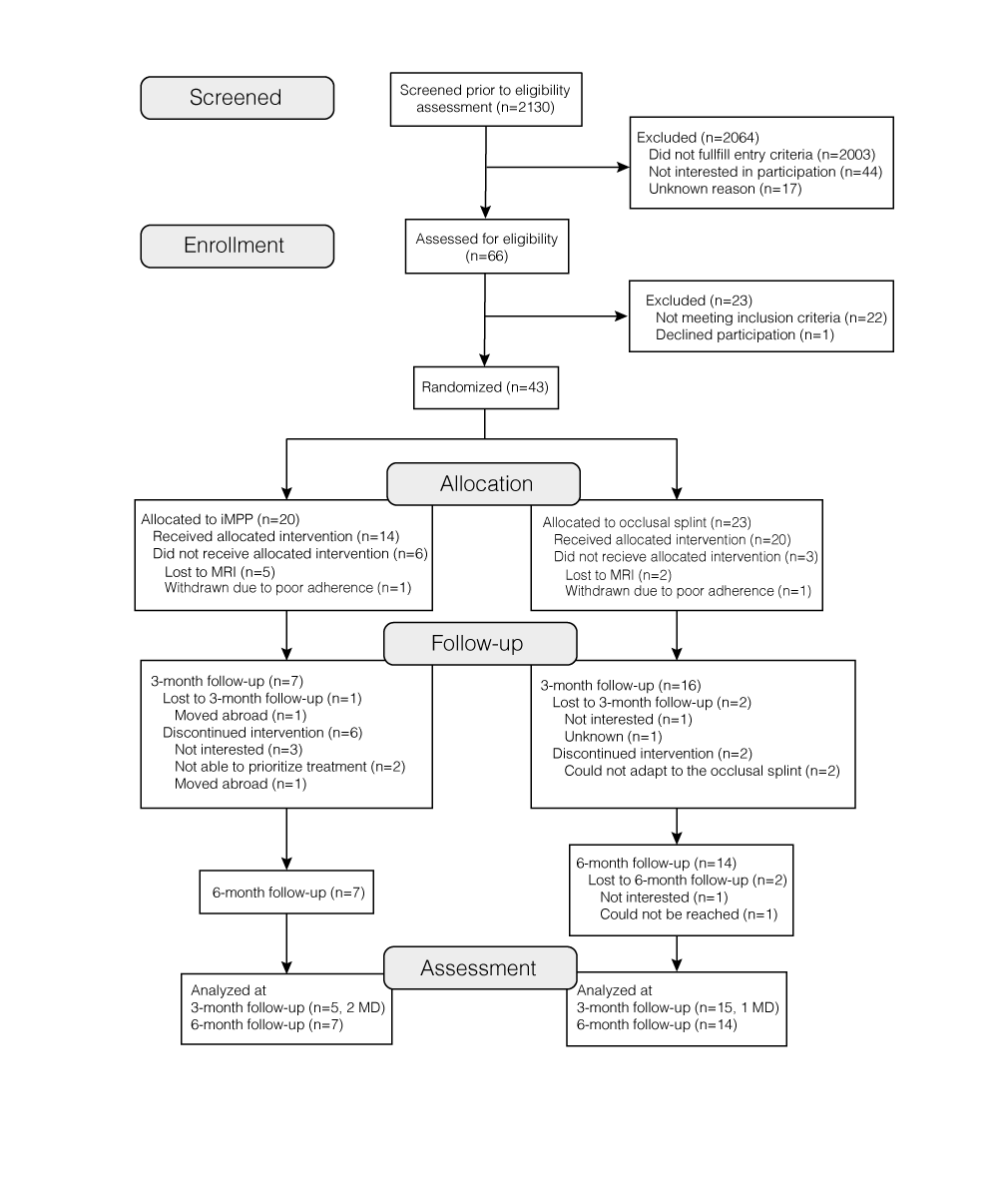
Flow diagram of the unblinded, parallel-arm, pilot randomized trial. iMPP: internet-based multimodal pain program; MRI: magnetic resonance imaging.

Participant flow through each stage of the trial is presented in Figure 2. The baseline characteristics of the sample are described in Table 2. Comparisons of baseline demographic, clinical, and psychosocial characteristics showed no statistical difference between treatment groups except that the occlusal splint group had a significantly greater number of jaw muscles with pain on palpation (*P=*.049; Table 2).

**Table 2:** Demographic, clinical, and psychosocial characteristics at baseline for patients with chronic temporomandibular disorder pain randomized to treatment with a guided internet-based multimodal pain program (iMPP, n=20) or occlusal splint (control, n=23).

Variable				iMPP^a^		Controls		*P* value
**Demographics**								
	Age (years), median (IQR)		27 (23-37)		27 (23-38)		.71	
	**Sex, n (%)**						.54	
		Male	5 (25)		4 (17)			
		Female	15 (75)		19 (83)			
	**Birth country, n (%)**						.76	
		Sweden or other Nordic country	18 (90)		20 (87)			
		Other	2 (10)		3 (13)			
	**Civil status, n (%)**						.09	
		Single	8 (40)		4 (17)			
		Married or de facto	6 (30)		14 (61)			
		Divorced	2 (10)		0 (0)			
		Other	4 (20)		5 (22)			
	**Education level, n (%)**						.43	
		University	9 (45)		12 (52)			
		Professional training	1 (5)		3 (13)			
		High school	10 (50)		7 (30)			
		Elementary school	0 (0)		1 (4)			
	**Employment status, n (%)**						.29	
		Employed	17 (85)		19 (83)			
		Unemployed	1 (5)		4 (17)			
		Retired	0 (0)		0 (0)			
		Registered disabled	1 (5)		0 (0)			
		Missing data	1 (5)		0 (0)			
**Clinical characteristics**								
	Characteristic pain intensity (0-10), median (IQR)		4.7 (3.7-5.3)		4 (3.7-5.7)		.61	
	Duration of temporomandibular disorder pain (months), median (IQR)		30 (12-120)		102 (12-300)		.36	
	Pain free mouth opening (mm), median (IQR)		41 (33-51)		36 (27-51)		.46	
	Maximum Unassisted mouth opening (mm), median (IQR)		52 (48-57)		49 (41-59)		.29	
	Number of muscles with pain on palpation (0-4), median (IQR)		4 (2-4)		4 (4-4)		.049	
	**Pain distribution, n (%)**						.74	
		Face	11 (55)		11 (48)			
		Face and neck	9 (45)		12 (52)			
		Other parts of the body	11 (55)		11 (48)			
	**Self-reported comorbidities, n (%)**						.40	
		Gastrointestinal disorders	5 (25)		1 (4)			
		Neurological disorder	1 (5)		0 (0)			
		Psychiatric disorder	6 (30)		3 (13)			
		Tinnitus	6 (30)		7 (30)			
		Other pain states	6 (30)		7 (30)			
**Psychosocial characteristics, median (IQR)**								
	Pain- related disability (GCPS DS^b^, 0-10)		0.8 (0.0-2.0)		0.3 (0.0-2.3)		.98	
	Awake parafunctional behaviors (OBC^c^, 0-76)		26 (21-31)		24 (19-30)		.71	
	Jaw function limitation (JFL^d^, 0-10)		0.5 (0-1.4)		0.8 (0.1-1.8)		.55	
	Depression (PHQ-9^e^, 0-27)		7 (3-11)		5 (2-10)		.52	
	Anxiety (GAD-7^f^, 0-21)		5 (2-8)		5 (2-9)		.52	
	Unspecific physical symptoms (PHQ-15^g^, 0-30)		8 (6-12)		8 (5-10)		.86	
	Stress (PSS-10^h^, 0-40)		13 (10-21)		12 (9-20)		.68	
	Catastrophizing (PCS^i^, 0-52)		16 (7-25)		12 (5-18)		.20	
	**Treatment expectation (SETS^j^** **, 1-7), n (%)**							
		Positive expectancy	5.3 (4.7-5.7)		5.0 (4.3-5.7)		.56	
		Negative expectancy	2.0 (2.0-3.3)		2.3 (2.0-3.0)		.81	

^a^iMPP: Internet-based multimodal pain program.

^b^GCPS DS: Graded Chronic Pain Scale Disability Score.

^c^OBC: Oral Behaviors Checklist.

^d^JFL: Jaw Functional Limitation Scale-8.

^e^PHQ-9: Patient Health Questionnaire-9.

^f^GAD-7: Generalized Anxiety Disorders Assessment -7.

^g^PHQ-15: Patient Health Questionnaire-15.

^h^PSS-10: Perceived Stress Scale-10.

^i^PCS: Pain Catastrophizing Scale.

^j^SETS: Stanford Expectations of Treatment Scale.

### Preliminary Evaluation of Participant Responses to Intervention

The between-group analysis of change in outcome measures (Table 3) showed no significant difference between the treatment groups at any follow-up regarding characteristic pain intensity (3 months: *P=*.58; 6 months: *P=*.41), pain-related disability (3 months: *P=*.51; 6 months: *P=*.12), jaw functional limitation (3 months: *P=*.45; 6 months: *P=*.90), degree of depression (3 months: *P=*.64; 6 months: *P=*.65), anxiety (3 months: *P=*.93; 6 months: *P=*.31), stress (3 months: *P=*.66; 6 months: *P=*.74), or catastrophizing (3 months: *P=*.86; 6 months: *P=*.85). Within-group analysis (Table 3) showed a significant reduction in jaw function limitation at the 6-month follow-up in the internet-based multimodal pain program group (Friedman: χ^2^_2_=10.2, *P=*.04; Wilcoxon: *z*=–2.3, *P=*.02). In the occlusal splint group, characteristic pain intensity was significantly reduced at both 3- and 6-month follow-up (Friedman: χ^2^_2_=25.1, *P=*.01; Wilcoxon 3 month: *z*=–3.0, *P=*.003; Wilcoxon 6 month: *z*=-3.3, *P=*.001) and jaw function limitation was reduced at the 6-month follow-up (Friedman: χ^2^_2_=20.0, *P=*.045; Wilcoxon: *z*=–2.33, *P=*.02).

**Table 3:** Difference in outcome measure scores at 3- and 6-month follow-up, as well as results from within- and between-group comparisons.

Outcomes^a^					Between-group differences						Within-group differences										
					Wilcoxon rank-sum						Friedman					Wilcoxon signed-rank					
			3 months^b^	6 months^c^	3 months			6 months									3 months			6 months	
					*z* score	*P* value	*z* score		*P* value	χ^2^			*P* value		*z* score			*P* value	*z* score		*P* value
**Primary outcomes**																					
	**ΔGCPS CPI^d^**				0.56	.58	0.82		.41												
		iMPP^e^	–1.3 (–2.0, –0.3)	–1.6 (–4.0, –0.3)						4.8			.31		N/A^f^			N/A	N/A		N/A
		Occlusal splint	–2.0 (–3.3, –1.0)	–2.5 (–3.3, –1.3)						25.1			.01		–3.01			.003	-3.27		.001
	**ΔGCPS DS^g^**				0.67	.51	1.55		.12												
		iMPP	0.0 (–1.0, 0.0)	0.0 (–0.3, 0.3)						5.5			.24		N/A			N/A	N/A		N/A
		Occlusal splint	–0.3 (–1.3, 0.0)	–0.7 (–2.0, 0.0)						20.2			.06		N/A			N/A	N/A		N/A
	**ΔJFL^h^**				–0.75	.45	0.12		.90												
		iMPP	–0.5 (–0.8, –0.3)	–0.5 (–0.9, –0.1)						10.2			.04		–1.91			.06	–2.29		.02
		Occlusal splint	0.0 (–0.9, 0.0)	–0.8 (–1.1, 0.0)						20.0			.045		–1.69			.09	–2.33		.02
**Secondary outcomes**																					
	**ΔPHQ-9^i^**				0.47	.64	–0.45		.65												
		iMPP	1.0 (–1.0, 2.0)	0.0 (–6.0, 2.0)						9.9			.04		0.54			.59	–0.69		.49
		Occlusal splint	0.0 (–3.0, 1.0)	–1.0 (–2.0, 2.0)						23.5			.02		–0.60			.55	–0.51		.61
	**ΔGAD-7^j^**				0.09	.93	–1.02		.31												
		iMPP	0.0 (0.0, 0.0)	0.0 (–6.0, 1.0)						9.9			.04		–0.16			.88	–0.95		.34
		Occlusal splint	–1.0 (–2.0, 3.0)	0.0 (–2.0, 1.0)						27.5			.007		0.11			.91	–0.16		.87
	**ΔPSS-10^k^**				0.44	.66	–0.34		.74												
		iMPP	4.0 (–2.0, 6.0)	0.0 (–8.0, 2.0)						10.9			.03		0.41			.69	–0.77		.44
		Occlusal splint	0.0 (–4.0, 5.0)	–0.5 (–5.0, 3.0)						27.9			.006		0.26			.80	–0.25		.80
	**ΔPCS^l^**				0.18	.86	0.19		.85												
		iMPP	–1.0 (–6.0, 0.0)	–4.0 (–6.0, 1.0)						9.5			.049		–0.54			.59	–1.02		.31
		Occlusal splint	–2.0 (–7.0, 0.0)	–3.0 (–11.0, 0.0)						20.8			.052		N/A			N/A	N/A		N/A

^a^Difference in outcome measure between follow-ups and baseline.

^b^At 3-month follow-up—internet-based multimodal pain program group: n=5 and occlusal splint group: n=15.

^c^At 6-month-follow up—internet-based multimodal pain program group: n=7 and occlusal splint group: n=14.

^d^GCPS CPI: Graded Chronic Pain Scale Characteristic Pain Intensity.

^e^iMPP: internet-based multimodal pain program.

^f^N/A: not applicable.

^g^GCPS DS: Graded Chronic Pain Scale Disability Score.

^h^JFL: Jaw Functional Limitation Scale-8.

^i^PHQ-9: Patient Health Questionnaire-9 (for depression).

^j^GAD-7: Generalized Anxiety Disorders-7.

^k^PSS-10: Perceived Stress Scale-10.

^l^PCS: Pain Catastrophizing Scale.

### Pretreatment Expectations

Comparison of pretreatment expectations on allocated treatment did not show any significant difference in positive (*P=*.56) or negative expectancy (*P=*.81) between groups (Table 2). In the internet-based multimodal pain program group there was a significant correlation between positive expectancy at baseline and change in pain-related disability (ρ=0.78, *P=*.04).

There was also a significant negative correlation between positive expectancy and change in the degree of depression (ρ=–0.78, *P=*.04). In the occlusal splint group, positive expectancy was positively correlated to change in catastrophizing (ρ=0.66, *P=*.01). No significant correlation was found between negative expectancy and change in treatment outcomes (Table 4).

**Table 4:** Spearman correlations for positive and negative treatment expectancy between baseline and difference in outcome measurements 6 months after treatment start.

Variable	Internet-based multimodal pain program (n=7)				Occlusal splint (n=14)			
	Positive expectancy		Negative expectancy		Positive expectancy		Negative expectancy	
	ρ	*P* value	ρ	*P* value	ρ	*P* value	ρ	*P* value
Characteristic pain intensity	0.26	.57	0.19	.69	0.25	.39	0.24	.40
Pain-related disability (GCPS DS^a^, 0-10)	0.78	.04	0.65	.11	0.49	.08	–0.21	.48
Jaw function limitation (JFL^b^, 0-10)	–0.36	.43	–0.11	.81	0.18	.56	0.14	.65
Depression (PHQ-9^c^, 0-27)	–0.78	.04	–0.54	.21	–0.15	.61	–0.12	.69
Anxiety (GAD-7^d^, 0-21)	–0.42	.34	–0.19	.68	–0.48	.08	–0.16	.58
Stress (PSS-10^e^, 0-40)	–0.24	.61	0.04	.93	0.17	.55	0.24	.41
Catastrophizing (PCS^f^, 0-52)	–0.40	.38	0.32	.49	0.66	.01	0.25	.39
Positive expectancy (SETS^g^, 1-7)	N/A^h^	N/A	0.20	.41	N/A	N/A	0.07	.77
Negative expectancy (SETS, 1-7)	0.20	.41	N/A	N/A	0.07	.77	N/A	N/A

^a^GCPS DS: Graded Chronic Pain Scale Disability Score.

^b^JFL: Jaw Functional Limitation Scale-8.

^c^PHQ-9: Patient Health Questionnaire-9.

^d^GAD-7: Generalized Anxiety Disorders -7.

^e^PSS-10: Perceived Stress Scale-10.

^f^PCS: Pain Catastrophizing Scale.

^g^SETS: Stanford Expectations of Treatment Scale.

^h^N/A: not applicable.

### Feasibility

**Figure 3:**
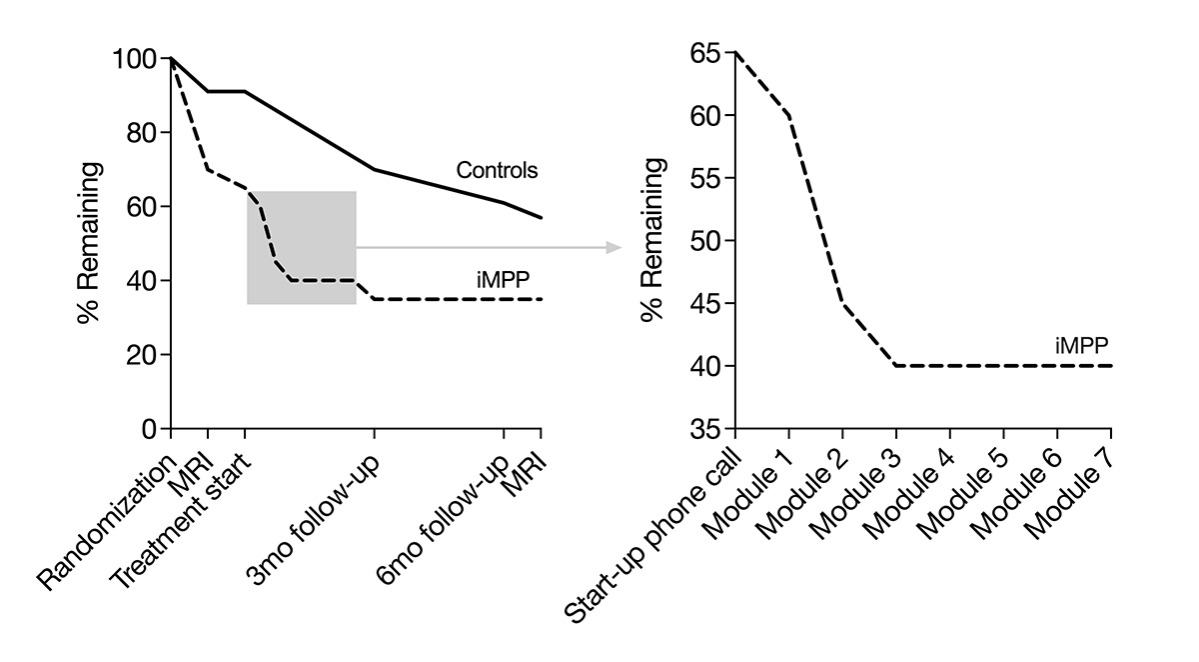
Attrition (left: the proportion of patients remaining in each treatment arm by event; right: an enlarged view of the proportion of internet-based multimodal pain program patients remaining by treatment module). iMPP: internet-based multimodal pain program; MRI: magnetic resonance imaging of the brain.

An average of 101 patients were screened per month, and 5 patients per month went through the inclusion examination. From this, 2 participants per month were enrolled, and 43 participants were recruited during the a priori determined recruitment period—fewer than had initially been planned. An attempt was made to extend the recruitment period, but it was considered futile under the current circumstances. The attrition rate showed an unbalanced dropout between groups (Figure 3). The proportion of dropout at the 6-month follow-up was 65% (13/20) for the internet-based multimodal pain program group and 39% (9/23) for the occlusal splint group. In total, 51% (22/43) of the enrolled patients did not complete the 6-month follow-up. Figure 3 also shows how many participants completed each module in the internet-based multimodal pain program treatment. Of the 20 participants in the internet-based multimodal pain program, 14 started treatment, and 8 completed all 7 modules of the program. Of those who completed the program, the time spent between the start-up phone calls and the last follow-up phone call was a median of 14 (IQR 8-15) weeks. No adverse events were reported by participants randomized to internet-based multimodal pain program, and none choose to receive additional treatment at the 3-month follow-up.

### Dropout Analysis

The dropout analysis showed no difference between completers and dropouts in the internet-based multimodal pain program group regarding demographic factors, clinical or psychosocial characteristics (Multimedia Appendix 3). In the occlusal splint group, the dropouts were significantly younger (*P=*.02), had a lower proportion of married or de facto (*P=*.01), a lower proportion of participants with full time employment (*P<*.01), and a higher number of unspecific physical symptoms compared to completers (*P=*.03; Multimedia Appendix 3).

### Posthoc Power

Posthoc power calculations showed a power of 11% and a sample size of 292 required for detecting a significant difference in characteristic pain intensity between 2 groups with α=.05 and β=.80 (characteristic pain intensity at the 6-month follow-up—internet-based multimodal pain program: mean 2.85; occlusal splint: mean 2.17; entire sample SD 1.92)

## Discussion

### General

This study set out to assess the effectiveness of an internet-based multimodal pain program for adults with chronic TMD pain in comparison to occlusal splint therapy in general dentistry. No differences in clinical outcomes were found between the 2 treatment modalities. However, within the internet-based multimodal pain program group, jaw functional limitation was reduced and within the occlusal splint group, characteristic pain intensity and jaw functional limitation was reduced. As the study proceeded, a substantially higher dropout rate than expected was observed. Consequently, this pilot study showed that a randomized controlled trial with this design is not feasible.

### No Difference in Treatment Effect

This study did not find any difference in any of the recommended IMMPACT core outcome measures between the internet-based multimodal pain program and occlusal splint therapy. One possible explanation for this is the small sample size due to fewer participants recruited than planned and the substantial dropout rate. The lack of sufficient power due to the small sample size is a major limitation of this study regarding interpretation of treatment effect. Another possible explanation is our choice to include an active control condition. In this study, the effectiveness of internet-based multimodal pain program was compared to occlusal splint therapy—a treatment with moderate efficacy on TMD pain [5]. Our choice of this control condition in an early phase trial can be debated. However, according to the Helsinki declaration, “The benefits, risks, burdens, and effectiveness of a new intervention must be tested against those of the best proven intervention(s), except in the following circumstances: where no proven intervention exists, the use of placebo, or no intervention, is acceptable [32].”

### The Internet-Based Multimodal Pain Program Improves Jaw Function

Physical function is an important factor to assess in chronic pain studies [28]. In this study, we used the Jaw Function Limitation scale to assess jaw function. Within-group analysis indicated that the internet-based multimodal pain program increases jaw function for those with chronic TMD pain. This can be expected because the internet-based multimodal pain program comprises jaw exercises as well as psychoeducation and situational analyses that aim to reduce fear of movement [33]. The multimodal internet-based pain program therapy, therefore, seems to improve physical function. However, the findings cannot be generalized due to the small sample size. Potential treatment effects on the other outcome measures were not possible to determine in this study. The lack of change regarding pain intensity and pain-related disability was somewhat unexpected, as others have shown that multimodal treatment and cognitive behavior therapy are effective for patients with chronic orofacial pain [34-36], and internet interventions have been shown to improve pain interference and pain intensity [9,37].

The IMMPACT core outcome domain of participant ratings of global improvement was not assessed in this study since it is not a part of the DC/TMD instrument that was used in this study. Future studies should consider assessing global change because it could provide an estimate of overall change in symptoms in addition to those of the primary outcomes. This may be especially important when investigating a multicomponent treatment package where the relative contribution of each active component is unknown.

### Occlusal Splint Treatment Reduces Pain and Improves Jaw Function

Within the occlusal splint group, characteristic pain intensity and jaw functional limitation were significantly reduced at the 6-month follow-up compared to baseline. The treatment effect of occlusal splint treatment on pain intensity was expected and is consistent with the literature [38]. A reduction in jaw functional limitation after occlusal splint therapy has also been reported [39]. It can, therefore, be assumed that the delivered control condition worked as expected.

### Pretreatment Expectation

IMMPACT recommends that expectations be considered as a phenotypic measure in clinical trials, and in this study, it was assessed with the multidimensional Stanford Expectations of Treatment Scale [40]. Research suggests that expectations influence the treatment outcome, and patients with positive expectations seem to benefit from medical treatment to a greater extent for a variety of conditions, including chronic pain [41]. The negative relation between positive expectancy and degree of depression in the internet-based multimodal pain program group is in line with this. Interestingly, this study also found a positive relation between positive expectancy and pain-related disability in the internet-based multimodal pain program group and that positive expectancy was positively associated with catastrophizing in the occlusal splint group. These relationships are interesting because it might suggest that positive expectations that are too high could have a negative impact on treatment outcome and also highlights the complex relationship of expectations on treatment and treatment outcome. Further studies are recommended to better understand the relationship between pretreatment expectations and outcomes of orofacial pain management protocols.

### Randomized Control Trial Not Feasible

Individuals with chronic TMD pain were effectively recruited with this study protocol. All eligible patients except one chose to participate in the study, suggesting that participation was appealing. Retaining participants, on the other hand, turned out to be more of a challenge.

The high proportion of loss to follow-up confirms the difficulty of collecting data in longitudinal studies. However, the total proportion of dropouts in this trial (22/43, 51%) was relatively high compared to those for other clinical chronic pain trials, for which attrition ranged from 5% to 46% [42]. It was also surprising that some participants dropped out before the treatment start. A possible explanation for this might be the mandatory MRI examinations before and after the treatment period, particularly given that the MRI examinations were performed in a different city than that in which the patients were recruited. In addition, technical issues with the MRI scanner during a 6-month period in 2017 caused further inconvenience for the participants.

In addition to high attrition, unbalanced dropout for the 2 treatment modalities at the 6-month follow-up was observed. This imbalance could be explained neither by a difference between the treatment groups regarding baseline characteristics, treatment expectations, adverse effects, or the need of rescue treatment nor by differences in baseline characteristics between dropouts and completers. The dropout rate in the occlusal splint group was 39% (9/23). A systematic review of randomized controlled trials on occlusal treatments in TMD reported a dropout rate of less than 10% [43]. We believe that the MRI examinations were a major factor in the unexpectedly high dropout rate in this group. In the internet-based multimodal pain program group, the dropout rate of 65% is comparable to those of other internet-based trials; attrition has been recognized as one of the methodological challenges in the evaluation of eHealth apps [44], and future studies should consider an allocation process that compensates for this risk. It is, however, clear that that a randomized controlled trial with the current design is not feasible from economic and ethical perspectives due to the high attrition rate, even if the study may be considered practically feasible.

### Future Studies

Further work is required to explore the potential of internet-based treatment for chronic orofacial pain. However, since the feasibility of this study design was suboptimal, different approaches have to be considered. For a future trial, adjustments in study design should be considered to reduce nonusage and dropout attrition. Inspired by a prior study [45] on an eHealth app for pain with low nonusage attrition, changes to the internet-based multimodal pain program could include optimization of scheme and triggers for reminders and offer different levels of clinical support according to the patient's preferences. Furthermore, the internet-based treatment used by that study had fewer modules that were less time consuming compared to those in this study's internet-based multimodal pain program [45]. Fewer and more focused modules should, therefore, be considered. Other possible changes to the internet-based multimodal pain program to make it more user-friendly include converting the offline workbook to a digital format and developing an app for mobile devices. In addition to improving the internet-based multimodal pain program itself, changes in the study protocol and design should be considered. We believe that the additional pre and posttreatment MRI examination was the primary reason for the high dropout rate in this study. Accordingly, a study of the treatment effect of internet-based multimodal pain program should not include MRI examinations. Also, conversion to a hybrid trial model with follow-up data collection online could make it more convenient to participate in the study and increase retention. Finally, novel study designs such as *n*-of-1 or noninferiority trials should be considered to optimize the quality and quantity of data acquired [46,47].

### Conclusion

This study was not able to demonstrate a difference in treatment outcome between an internet-based multimodal pain program and occlusal splint therapy in patients with chronic TMD pain. However, within the internet-based multimodal pain program group, the results suggested that internet-based multimodal pain program improves jaw function. The results also confirmed the treatment effect of occlusal splint therapy in chronic TMD pain. Furthermore, the outcome of this pilot study showed that a randomized controlled trial with this design is not feasible due to a too high attrition rate.

## Appendix

Multimedia Appendix 1 Informed consent documentation.

Multimedia Appendix 2 Demo information.

Multimedia Appendix 3 Table of baseline characteristics of dropouts and completers of the sample (patients with chronic temporomandibular pain, n=43) within the allocated treatment groups.

## Abbreviations

DC/TMD: Diagnostic Criteria for Temporomandibular Disorders
IMMPACT: Initiative on Methods, Measurement, and Pain Assessment in Clinical Trials
MRI: magnetic resonance imaging
TMD: temporomandibular disorders
