# Supplementary material for: Internet-Based Multimodal Pain Program With Telephone Support for Adults With Chronic Temporomandibular Disorder Pain: Randomized Controlled Pilot Trial
Source: J Med Internet Res. 2020 Oct 13;22(10):e22326. doi: 10.2196/22326 (PMC7592067; doi:10.2196/22326)
Supplement: Multimedia Appendix 1 [file jmir_v22i10e22326_app1.pdf]

Multimedia Appendix 1. Informed consent documentation

FORSKNINGSPERSONSINFORMATION

Kan internet-baserad beteendeterapi i allmäntandvården återställa hjärnans funktion och morfologi vid kronisk käksmärta hos vuxna?

1. Bakgrund och syfte

Forskningsprojektet avser att undersöka om en internetbaserad smärtskola är en bättre behandling än bettskena (konventionell behandling) i allmäntandvård för vuxna patienter med kronisk käksmärta. Projektet kommer även undersöka om denna smärtskola kan återställa funktionen i hjärnans smärtcentra och vad som i så fall kan förutsäga denna effekt.

Mellan 5 och 14% av befolkningen har kronisk käksmärta och förekomsten är högst bland kvinnor i arbetsför ålder. Den vanligaste orsaken till denna kroniska smärta är smärta och käkfunktionsstörningar i ansiktets och huvudets muskulatur, leder, nerver, kärl och bindväv. Det vanligaste symtomet är smärta i tuggmuskulatur och käkledsregionerna, det vill säga i tinningar, ansikte och områdena framför öronen. Smärtan förvärras ofta vid käkrörelser.

Kronisk smärta bör betraktas och behandlas som en sjukdom i sig. Smärtupplevelsen uppkommer genom aktivitet i tre centra i hjärnan: känselcentrum som talar om var smärtan är lokaliserad, hur intensiv den är och vilken karaktär den har; känslocentrum som påverkas av och påverkar smärta via depression, oro och rädsla samt förståelsecentrum som påverkar via förväntningar, tidigare erfarenheter, sammanhang osv. Aktiviteten i dessa centra är förhöjd hos patienter med kronisk smärta, avsevärt mer i känslo- och förståelsecentra än i känselcentrum. Det finns alltså mätbara förändringar i hjärnans smärtsystem när smärtan blir kronisk.

Beteendepåverkande (smärtskola) behandling har vistats vara effektiv för att minska kronisk smärta. Beteendeterapi är patientaktiverande och kan omfatta patientundervisning, avslappningsövningar, rörelseövningar och smärt- och stresshantering.

2. Förfrågan om deltagande

Du tillfrågas att vara med i denna studie då du möjligen kan ha käksmärta enligt screeningfrågorna vi ställt till dig.

3. Hur går studien till?

Studien omfattar 60 patienter med käksmärta och 30 friska.

Studien innehåller 1) frågeformulär (<30 min) samt strukturerad undersökning av käksystemet hos din tandläkare (ca 15 min), undersökning i magnetkamera (MR; 45 min), 3) behandling med antingen internet-baserad smärtskola eller bettskena, 4) uppföljning via hemskickade frågeformulär

efter 3 och 12 månader, 5) förnyad MR-undersökning.

Vid uppföljning efter 3 mån kommer de patienter som upplever behov att få extra utredning och behandling på specialistavdelningen för Orofacial smärta och käkfunktion, Malmö. Dessa utgår då ur studien.

5. Vilka är riskerna?

Komplikationer kan vara en kortvarig smärta vid lätt tryck mot käkmuskulatur eller käkled under och efter undersökningen.

Dessa eventuella komplikationer är desamma i denna studie som vid den utredning som rutinmässig genomförs vid specialistkliniken, d.v.s. projektet innebär i sig ingen ytterligare risk för komplikationer.

Riskerna med att vara med i studien är små, med få förväntade komplikationer och dessa är, om de förkommer, av lindriga och snabbt övergående karaktär.

Den förväntade nyttan med studien överväger de mycket lindriga och sällsynta besvär som kan uppkomma.

Eventuella patologiska fynd vid MR-undersökningen kommer att snarast efter granskning meddelas patienten som erbjuds remiss till utredning och eventuell behandling.

6. Finns det några fördelar?

Internet-baserad beteendeterapi har visats vara effektiv behandlingsmetod mot många tillstånd, t ex generell smärta, nedstämdhet och oro. Effekt på käksmärta är ännu okänd men det finns anledning att anta att den ska kunna ha effekt på käksmärta. Bettskena har visats ge smärtlindrande effekt på käksmärta. Detta innebär att deltagande i studien kan ha omedelbar nytta för deltagande patienter förutom en möjlig nytta för kommande patienter efter projektets genomförande.

7. Hantering av data och sekretess

Undersökningsdata sparas i forskningsformulär som förvaras inlåst vid avdelningen Orofacial smärta och käkfunktion eller Orofaciala smärtenheten, Malmö. MR-data kommer även att hanteras i de system som Bild- och funktionsdiagnostik vid Skånes Universitetssjukhus använder rutinmässigt för deras MR-undersökningar. Patienter registreras med personnummer i patientjournalen men anonymiserat med forskningspersonnummer i forskningsformulären. Tillgång till låst skåp med kodlista har endast projektansvarig och medverkande forskare och doktorander. Forskningsformulären sparas för att säkra framtida tillgång till rådata.

8. Information angående behandling av personuppgifter

De uppgifter som finns registrerade om dig är sekretesskyddade och kommer att behandlas så att inte obehöriga kan ta del av dem. Hanteringen av dina uppgifter regleras av Personuppgiftslagen (PuL, SFS:1998:204). Ansvarig för dina studiedata är huvudmannen för

forskningen, Malmö högskola. Rektor för Malmö Högskola är personuppgiftsansvarig och företräds av personuppgiftsombud Hans Jonsson, 0708-655289.

De journaluppgifter som insamlas kommer att överföras till ett register i anonymiserad form. Ändamålet med registret är att sammanställa och statistiskt behandla insamlade journaluppgifter i det vetenskapliga arbetet.

De personuppgifter som ingår i registret är ålder och kön samt uppgifter från den kliniska undersökningen och frågeformuläret. Inga andra uppgifter kommer att insamlas.

Du har rätt att ta del av de uppgifter som finns registrerade om dig efter en skriftlig ansökan till ansvarig forskare (Malmö Högskola, Odontologiska fakulteten, Per Alstergren, 205 06 Malmö). Du har också rätt till korrigering av eventuella oriktiga eller missvisande uppgifter gällande dig i registret. Dina svar och dina resultat kommer att förvaras och behandlas så att inte obehöriga kan ta del av dem.

Resultaten redovisas på gruppnivå. Innan databearbetningen kommer all data vara anonym genom att kodnummer för varje forskningsperson används istället för personnummer och namn.

De studier som projektet ger upphov till kommer att publiceras i internationella vetenskapliga tidskrifter och kommuniceras vid internationella forskningskonferenser.

9. Hur får jag information om studiens resultat?

Resultaten kommer att publiceras på gruppnivå i internationella vetenskapliga tidskrifter.

10. Försäkring, ersättning

Alla forskningspersoner omfattas av patientförsäkringen. Ingen ersättning betalas ut.

11. Frivillighet

Deltagande i detta forskningsprojekt är frivilligt och man har när som helst, utan särskild förklaring och utan konsekvenser, rätt att avbryta. Att avbryta studien påverkar inte behandling eller övrigt omhändertagande.

11. Ansvariga

Forskningshuvudman för detta forskningsprojekt är Malmö högskola. Företrädare för forskningshuvudmannen och huvudansvarig för genomförande för projektet är:

Per Alstergren
Docent, övertandläkare

Malmö högskola
Odontologiska fakulteten
Orofaciala smärtenheten
205 06 Malmö
Tel: 0725-310067
per.alstergren@mah.se
Ansvarig för patientbehandling och forskningsundersökning:

Per Alstergren
Docent, övertandläkare

Malmö högskola
Odontologiska fakulteten
Orofaciala smärtenheten
205 06 Malmö
Tel: 0725-310067
per.alstergren@mah.se

Ansvarig inom Folktandvården Skåne AB:

Marika Qvist
VD
Folktandvården Skåne AB
Huvudkontoret
Dockplatsen 1
211 19 Malmö
