# Supplementary material for: Internet-Based Multimodal Pain Program With Telephone Support for Adults With Chronic Temporomandibular Disorder Pain: Randomized Controlled Pilot Trial
Source: J Med Internet Res. 2020 Oct 13;22(10):e22326. doi: 10.2196/22326 (PMC7592067; doi:10.2196/22326)
Supplement: Multimedia Appendix 2 [file jmir_v22i10e22326_app2.pdf]

A demo of the internet-based multimodal pain program is available at:

https://program.kbtonline.se

Username: Demo_Smartskola_Vuxna
Password: 27-M-63-DW

This is a Multimedia Appendix to a full manuscript published in the J Med Internet Res. For full copyright and citation information see http://dx.doi.org/10.2196/jmir.22326
