# Supplementary material for: Internet-Based Multimodal Pain Program With Telephone Support for Adults With Chronic Temporomandibular Disorder Pain: Randomized Controlled Pilot Trial
Source: J Med Internet Res. 2020 Oct 13;22(10):e22326. doi: 10.2196/22326 (PMC7592067; doi:10.2196/22326)
Supplement: Multimedia Appendix 3 [file jmir_v22i10e22326_app3.pdf]

Multimedia Appendix 3.

Table of baseline characteristics of dropouts and completers of the sample within the allocated treatment groups, internet-based multimodal pain program (iMPP) or occlusal splint.

	iMPP			Occlusal splint		
**Variable**	Completers (n=7)	Dropouts (n=13)	***P***	Completers (n=14)	Dropouts (n=9)	***P***
**Demographics**						
Age (years), median (IQR)	23 (21-31)	27 (26-37)	.23	37 (23-47)	26 (20-26)	**.02**
**Sex, n (%)**			.79			.62
Male	2 (29)	3 (23)		2 (14)	2 (22)	
Female	5 (71)	10 (77)		12 (86)	7 (78)	
**Birth country, n (%)**			.64			.14
Sweden or other Nordic country	6 (86)	12 (92)		11 (79)	9 (100)	
Other	1 (14)	1 (8)		3 (21)	0(0)	
**Civil status, n (%)**			.37			**.01**
Single	4 (57)	4 (31)		3 (21)	1 (11)	
Married or de facto	1 (14)	5 (38)		11 (79)	3 (33)	
Separated/divorced/widowed	0 (0)	2 (15)		0 (0)	0 (0)	
Other	2 (29)	2 (15)		0 (0)	5 (56)	
**Education level, n (%)**			.06			.31
University	1 (14)	8 (62)		9 (64)	3 (33)	
Professional training	0 (0)	1 (8)		1 (7)	2 (22)	
High school	6 (86)	4 (31)		4 (29)	3 (33)	
Elementary school	0 (0)	0 (0)		0 (0)	1 (11)	
**Employment status, n (%)**			.60			**<.01**
Employed	7 (100)	10 (77)		14 (100)	5 (56)	
Unemployed	0 (0)	1 (8)		0 (0)	4 (44)	
Retired	0 (0)	0 (0)		0 (0)	0 (0)	
Registered disabled	0 (0)	1 (8)		0 (0)	0 (0)	
Missing data	0 (0)	1 (8)		0 (0)	0 (0)	
**Clinical characteristics**						
Characteristic pain intensity (0-10), median (IQR)	4.3 (3.0-5.3)	4.7 (4.3-5.3)	.43	4.0 (3.7-5.3)	4.3 (3.7-5.7)	.82
Duration of temporomandibular disorder pain (months), median (IQR)	12 (12-30)	69 (18-138)	.24	180 (60-360)	42 (9-180)	.11
Pain free mouth opening (mm), median (IQR)	36 (22-50)	36 (30-45)	.87	34 (25-50)	32 (21-39)	.43
Maximum Unassisted mouth opening (mm), median (IQR)	39 (38-55)	50 (45-54)	.18	44 (38-56)	45(43-50)	.92
Number of muscles with pain on palpation (0-4), median (IQR)	3 (2-4)	4 (2-4)	.90	4 (4-4)	4 (4-4)	.93
**Pain distribution, n (%)**			.56			.91
Face	5 (71)	6 (46)		7 (50)	4 (44)	
Face and neck	2 (29)	7 (54)		7 (50)	5 (56)	
Other parts of the body	4 (57)	7 (54)		6 (43)	5 (56)	
**Self-reported comorbidities, n (%)**			.41			.70
Gastrointestinal disorders	3 (43)	2 (15)		1 (7)	0 (0)	
Neurological disorder	1 (14)	0 (0)		0 (0)	0 (0)	
Psychiatric disorder	2 (29)	4 (31)		1 (7)	2 (22)	
Tinnitus	1 (14)	5 (38)		3 (21)	4 (44)	
Other pain states	3 (43)	3 (23)		3 (21)	4 (44)	
**Psychosocial characteristics, median (IQR)**						
Pain- related disability (GCPS DS[a], 0-10)	0.3 (0.0-1.0)	2.0 (0.0-2.0)	.09	0.7 (0.0-2.0)	0.3 (0.0-2.3)	.95
Awake parafunctional behaviors (OBC[b], 0-76)	23 (18-34)	26 (25-31)	.61	23 (19-28)	29 (20-33)	.34
Jaw function limitation (JFL[c], 0-10)	1.3 (0.4-1.9)	0.3 (0.0-0.9)	.14	0.9 (0.1-1.5)	0.4 (0.0-2.0)	.95
Depression (PHQ-9[d], 0-27)	4 (0-16)	7 (4-10)	.72	4 (2-6)	10 (4-10)	.11
Anxiety (GAD-7[e], 0-21)	4 (2-9)	6 (4-8)	.55	4 (2-6)	5 (2-10)	.46
Unspecific physical symptoms (PHQ-15[f], 0-30)	6 (3-8)	8 (6-12)	.21	7 (4-8)	10 (9-14)	**.03**
Stress (PSS-10[g], 0-40)	14 (7-22)	12 (11-20)	.63	12 (7-19)	16 (11-22)	.28
Catastrophizing (PCS[h], 0-52)	16 (4-22)	15 (11-26)	.47	11 (5-18)	13 (11-27)	.31
**Treatment expectation (SETS[i], 1-7), n (%)**						
Positive expectancy	5.0 (4.7-5.7)	5.7 (4.7-6.0)	.44	5.2 (4.3-6.0)	5.0 (4.3-5.3)	.15
Negative expectancy	1.3 (1.0-3.0)	1.0 (1.0-2.0)	.60	1.2 (1.0-2.3)	1.3 (1.0-2.0)	.97

[a]GCPS DS: Graded Chronic Pain Scale Disability Score.

[b]OBC: Oral Behaviors Checklist.

[c]JFL: Jaw Functional Limitation Scale-8.

[d]PHQ-9: Patient Health Questionnaire-9.

[e]GAD-7: Generalized Anxiety Disorders Assessment -7.

[f]PHQ-15: Patient Health Questionnaire-15.

[g]PSS-10: Perceived Stress Scale-10.

[h]PCS: Pain Catastrophizing Scale.

[i]SETS: Stanford Expectations of Treatment Scale.

This is a Multimedia Appendix to a full manuscript published in the J Med Internet Res.
For full copyright and citation information see http://dx.doi.org/10.2196/jmir.22326
